# Rhenium (I) Complexes as Probes for Prokaryotic and Fungal Cells by Fluorescence Microscopy: Do Ligands Matter?

**DOI:** 10.3389/fchem.2019.00454

**Published:** 2019-06-26

**Authors:** Carolina Otero, Alexander Carreño, Rubén Polanco, Felipe M. Llancalahuen, Ramiro Arratia-Pérez, Manuel Gacitúa, Juan A. Fuentes

**Affiliations:** ^1^Facultad de Medicina, Escuela de Química y Farmacia, Universidad Andres Bello, Santiago, Chile; ^2^Center for Applied Nanosciences (CANS), Universidad Andres Bello, Santiago, Chile; ^3^Facultad de Ciencias de la Vida, Centro de Biotecnología Vegetal, Universidad Andres Bello, Santiago, Chile; ^4^Facultad de Química y Biología, Universidad de Santiago de Chile (USACH), Santiago, Chile; ^5^Laboratorio de Genética y Patogénesis Bacteriana, Facultad de Ciencias de la Vida, Universidad Andres Bello, Santiago, Chile

**Keywords:** rhenium (I) tricarbonyl complexes, Equatorial ligand, ancillary ligand, bacteria, fungi, yeasts, molds

## Abstract

Re(I) complexes have exposed highly suitable properties for cellular imaging (especially for fluorescent microscopy) such as low cytotoxicity, good cellular uptake, and differential staining. These features can be modulated or tuned by modifying the ligands surrounding the metal core. However, most of Re(I)-based complexes have been tested for non-walled cells, such as epithelial cells. In this context, it has been proposed that Re(I) complexes are inefficient to stain walled cells (i.e., cells protected by a rigid cell wall, such as bacteria and fungi), presumably due to this physical barrier hampering cellular uptake. More recently, a series of studies have been published showing that a suitable combination of ligands is useful for obtaining Re(I)-based complexes able to stain walled cells. This review summarizes the main characteristics of different fluorophores used in bioimage, remarking the advantages of d^6^-based complexes, and focusing on Re(I) complexes. In addition, we explored different structural features of these complexes that allow for obtaining fluorophores especially designed for walled cells (bacteria and fungi), with especial emphasis on the ligand choice. Since many pathogens correspond to bacteria and fungi (yeasts and molds), and considering that these organisms have been increasingly used in several biotechnological applications, development of new tools for their study, such as the design of new fluorophores, is fundamental and attractive.

## Cell Imaging Methods

Cell imaging has become a powerful tool to reveal particular biological structures and explore molecular mechanisms, unraveling dynamics, and functions of many different cellular processes (Rabuka et al., 2008; Hensle and Blum, 2013; Hananya et al., 2016; Majumder et al., 2016; Cui et al., 2017; Yoshimura, 2018). Accordingly, development of diverse transmitted light microscopy approaches, including fluorescence microscopy, is increasingly contributing to improve this technique (Roeffaers et al., 2008; Hauser et al., 2017). Fluorescence microscopy has been considered to be one of the most important advances to observe biological structures, but also to explore physiological processes or even characterize new compartments (Phimphivong and Saavedra, 1998; Bullok et al., 2002; Heintzmann and Huser, 2017). In this sense, research about new, improved fluorescent indicators (simply known as fluorophores) clearly constitutes a new challenge (Frederiksen et al., 2016; Yang et al., 2016; Xue et al., 2017; Bourassa et al., 2018).

Fluorescence microscopy relies upon the use of fluorescent agents, or those parts of a sample that are naturally emissive, to generate a detectable emitted light signal upon excitation (Borman, 2010; Vendrell et al., 2012; Kim et al., 2015; Tian et al., 2017). This allows for greater contrast between sections of the specimen and greater signal-to-noise ratios than conventional microscopy, which uses detection of reflected or transmitted light (Cheng et al., 2017; Gao et al., 2017; More et al., 2018).

Nevertheless, overall limitations of fluorescence microscopy, as an imaging technique, include low resolution to approximately half the wavelength of the light involved in the experiment, and the limited depth of tissue penetration of the light used. This limits visible light fluorescence microscopy to sample depths of a few millimeters and near IR microscopy to a few centimeters (Li et al., 2017; Taylor et al., 2018). For these reasons, it is necessary to develop new and improved luminescent fluorophores, suitable to be used with fluorescence microscopy.

## Luminescent Markers for Fluorescence Microscopy

In fluorescence microscopy, image quality depends largely on the physicochemical properties of the luminescent marker. For these reasons, markers should be carefully chosen to fulfill the requirements of a particular technique (Shaner, 2014). It has been considered that a good luminescent marker for imaging applications must exhibit some desirable properties, such as good stability and solubility in aqueous solvents (including buffer and culture media); low cytotoxicity, including low phototoxicity (i.e., toxicity generated upon light exposure) (Haas et al., 2014); differential affinity (i.e., specificity) for certain cell structures; and an efficient cellular uptake, hopefully in absence of other chemical or physical agents that artificially increase membrane permeability (Fernandez-Moreira et al., 2010). Other photophysical properties are also important. Accordingly, luminescent fluorophores must exhibit an efficient sample penetration to create high quality images. For instance, fluorophores with red shifted emission and excitation profiles, particularly in the near-infrared region, have shown a suitable penetration for biological systems (Zhao et al., 2014). In addition, luminescent markers should be easily distinguishable from the background, showing high brightness. In this context, background autofluorescence from biological systems generally reduce resolution of a luminescent marker. Since autofluorescence, produced by DNA, NADPH, and other biomolecules, normally presents small Stokes shift (Santoro et al., 2012; Balasubramaniam et al., 2015; Coda et al., 2015), it is desirable that luminescent markers present large Stokes shift, a property that contributes to preventing self-quenching (i.e., dimmer images) (Moriarty et al., 2014). Finally, markers must also show a relatively long luminescent lifetime (τ, from ~10^2^ to 10^6^ ms), a useful feature that also contributes to distinguish the desired signal from the biological system, which shows mostly short-lived autofluorescence (~10 ns). Thus, since different cellular structures present different τ with respect to autofluorescence, it is possible to remove this autofluorescence background or even use it to provide further information through fluorescence lifetime imaging microscopy/mapping (FLIM) (Coda et al., 2015).

At present, a wide variety of fluorophores for bioimaging applications have been reported. Among these, genetically encoded fluorescent proteins (FPs) (Enterina et al., 2015); organic dyes; quantum dots (Baker, 2010; Doane and Burda, 2012); and metal-based systems (Shang et al., 2011; Echeverría et al., 2012) are the most important.

### Fluorescent Proteins (FPs)

A wide variety of fluorescent proteins have been engineered, with several adaptations and characteristics to suit diverse applications, including presentation in different colors, from blue to far-red. FPs can be genetically encoded, a major advantage over other systems, thereby allowing direct labeling of many proteins in a living cell; albeit this advantage must be contrasted against poor quantum yield, low photon yield (i.e., FPs exhibit poor brightness), and a large size that impairs cellular uptake when they are heterologously produced, particularly in prokaryotic cells (bacteria) and eukaryotic walled cells such as yeasts or molds (Baird et al., 2000; Kubitscheck et al., 2000; Shaner et al., 2004). Moreover, not all FPs are stable; some of them exhibiting high degradation rates (Haas et al., 2014). Even more so, several chimeric proteins containing an FP moiety lack their original functions and/or do not exhibit luminescence due to inappropriate protein folding (Stepanenko et al., 2013). Another consideration when using FPs is the maturation time, i.e., time necessary to properly fold and emission. Typical maturation times are around 40 min, but depending on pH, temperature, and the specific FP, some may take several hours to mature (Baird et al., 2000; Shaner et al., 2004; Chudakov et al., 2010). Moreover, it is important to remark that the chromophore formation step requires the presence of oxygen in many FPs (including green fluorescence protein GFP), making these markers incompatible with obligate anaerobes (Haas et al., 2014). Furthermore, it is important to consider that the use of FPs is restricted to cellular systems that have well-established transformation protocols or availability of appropriate expression vectors.

### Organic Molecules

Most fluorophores used in confocal microscopy are organic molecules, normally a series of fused, heterocyclic rings (Wood, 1994). While extinction coefficients and quantum yield of many of these fluorophores are high, they exhibit small Stokes shift, and short luminescence lifetime compared to metal-based systems (see below). Unlike FPs, organic dyes are not genetically encodable, consequently they must be incorporated into the cell through the plasma membrane by diffusion, endocytosis or microinjection. Nevertheless, not all organic dyes can permeate cell membranes. For instance, rhodamine dyes can poorly diffuse across bacterial membranes, while sulfonated cyanine dyes are completely unable to enter into bacterial cells (Fernandez-Suarez and Ting, 2008). This is an especially critical point since endocytosis and microinjection are not available for prokaryotic cells. Instead, membrane permeabilization is normally used; albeit this procedure can produce misleading or confusing alterations in data (artifacts) due to the presence of organic solvents affecting membranes and other cell structures (Sochacki et al., 2011). In addition, considering that organic dyes usually bind non-specifically in the cell, most staining is limited regarding their specificity, needing several washing steps to remove excess, unbound dye from the cell (Fernandez-Suarez and Ting, 2008).

### Quantum Dots

Quantum dots have also been used for fluorescence microscopy, although they are much less common in applications due to their usual large size, requirement for surface passivation, and unpredictable blinking properties (Antelman et al., 2009; Wang et al., 2009; Mutavdzic et al., 2011; Ritchie et al., 2013). As well as with exogenous proteins and organic dyes, many quantum dots are difficult to be incorporated into cells, restricting their use to the outer membrane and cell surface in prokaryotic cells (Chalmers et al., 2007; Zhang et al., 2011; Ritchie et al., 2013). Although quantum dots commonly exhibit much longer photobleaching lifetimes compared to FPs, the presence of blinking constitutes a clear disadvantage to obtain high quality images (Michalet et al., 2005; Mahler et al., 2008; Omogo et al., 2016; Osborne and Fisher, 2016). Furthermore, another important disadvantage is the high toxicity of the semiconducting materials used in the fabrication of quantum dots like CdQ (Q = Se or Te) (Khalili Fard et al., 2015; Li et al., 2016; Silva et al., 2016). To address this issue, numerous studies have explored the development of non-toxic quantum dots. Nevertheless, only less-toxic quantum dots have been produced (Das and Snee, 2016). For that reason, they must be coated with organic molecules, a complex, and expensive procedure, to render them soluble and biocompatible by preventing the leaching of toxic ions (Fernandez-Moreira et al., 2010). Even though extinction coefficients and quantum yield of many quantum dots are high, these fluorophores exhibit normally smaller Stokes shift, shorter luminescence lifetime and higher susceptibility to photobleaching compared with metal-based systems (see below).

### Metal-Based Systems

Metal-based systems are comparatively smaller (1–2 nm), and often possess excellent optical properties such as high brightness, narrow emission bands, multiple emission wavelengths, emission tunability, long fluorescence lifetime, large Stokes shift, resistance to photobleaching, and high stability, compared with other fluorophores (Baird et al., 2000; Shaner et al., 2004; Ranjan et al., 2015). Due to all these properties, especially small size, the position of these dyes in a sample can be determined with high precision, a useful feature to perform super-resolution microscopy (Thompson et al., 2002; Agrawal et al., 2013; Haas et al., 2014). Some complexes of certain 4f elements (i.e., lanthanides) show extremely long luminescence lifetime (10^6^ ns) and, in some cases, they emit in the NIR region of the spectrum, which are features that make them attractive targets for applications in fluorescence microscopy (Song et al., 2008; Montgomery et al., 2009; Amoroso and Pope, 2015). Despite all these promising advantages, the presence of an additional chromophore must be incorporated into the complex (i.e., an antenna) to allow sufficient absorption and subsequent transfer of energy to the lanthanide; in other words, lanthanide ions are difficult to excite (Liu et al., 2013). In addition, lanthanide complexes must exhibit high stability to avoid the release of highly toxic lanthanide ions, a process demanding the synthesis of intricate macrocyclic ligands (Montgomery et al., 2009). Besides 4f elements (lanthanides), d^6^ metal-based systems, in combination with a relatively high amount of ligand-field dinitrogenated and/or organometallic ligands, present attractive features to be used as fluorophore for applications in fluorescence microscopy.

## d^6^ Metal-Based Complexes

Over the last few years, luminescent d^6^ complexes have attracted considerable interest for applications in microscopy as synthetic fluorescent dyes, mainly due to their attractive photophysical properties (Haas and Franz, 2009; Patra and Gasser, 2012). Typical d^6^ complexes of Re(I), Ru(II), Os(II), and Ir(III) (Lee et al., 2017), in combination of a relatively high diversity of ligands (e.g., ruthenium trisbipyridyls, rhenium *fac* tricarbonyl polypyridyls, osmium bipyridyl, and iridium cyclometallates complexes), have been used as fluorophores for fluorescent microscopy and related applications (Table 1) (Virel et al., 2009; Langdon-Jones et al., 2014; You et al., 2014; Gupta et al., 2016). In general, d^6^ complexes share common features making them suitable for microscopy applications. For instance, as well as luminescent lanthanide complexes, d^6^ complexes have large Stokes shift (hundreds of nm), which allow clear differentiation between autofluorescence and signal luminescence; long excited-state lifetimes, which can permit elimination of short-lived autofluorescence (ns) (Fernandez-Moreira et al., 2010; Li et al., 2011); enhanced photostability (leading to lower photobleaching) (Stufkens and Vlcek, 1998; Lowry et al., 2004); high chemical stability; and cellular uptake, at least for eukaryotic non-walled cells (Haas and Franz, 2009; Langdon-Jones et al., 2014).

**Table 1:** Examples of d^6^ complexes used as fluorophores in biological applications.

**Complex**	**Structure**	**Comment**	**References**
Re(I)	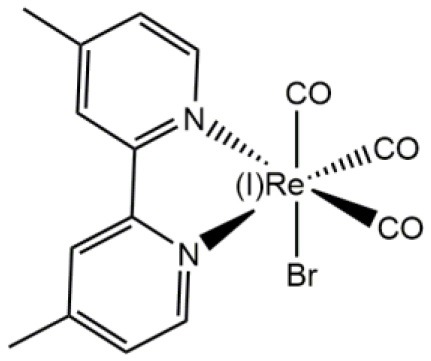	Soft staining of walled cells (yeasts, *Candida albicans*, and *Cryptococcus* spp.)	Carreño et al., 2017a
	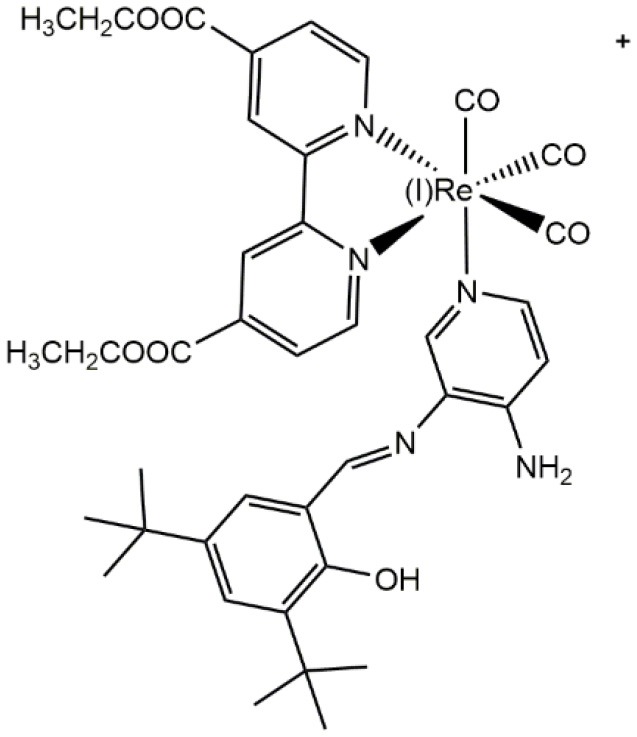	Differential staining of the nucleus in walled cells (yeasts, *Candida albicans*, and *Cryptococcus* spp.)	Carreño et al., 2015b, 2017a
Ru(II)	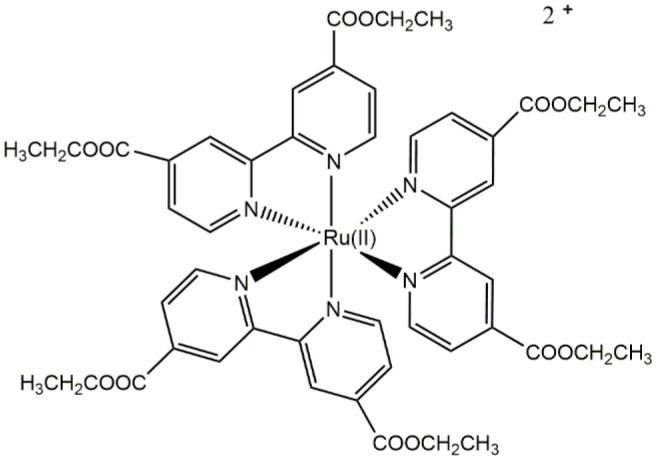	Differential staining of a structure presumably corresponding to the nucleus in walled cells (yeasts, *Candida albicans*)	Carreño et al., 2019b
	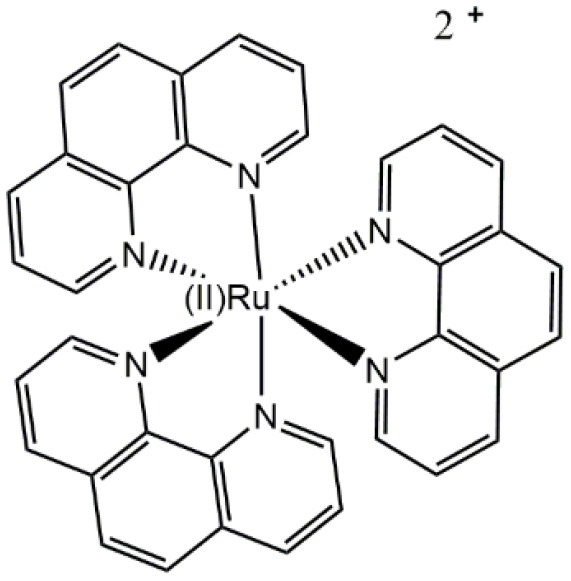	Differential staining of a structure corresponding to the cell envelop (presumably the cell wall) in walled cells (yeasts, *Candida albicans*)	Carreño et al., 2019b
Os(II)	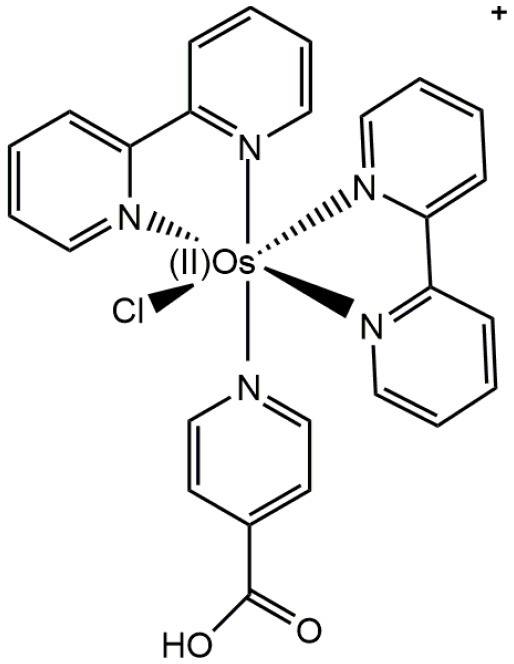	Universal luminescent probe for enzymatic reactions	Virel et al., 2009
Ir(III)	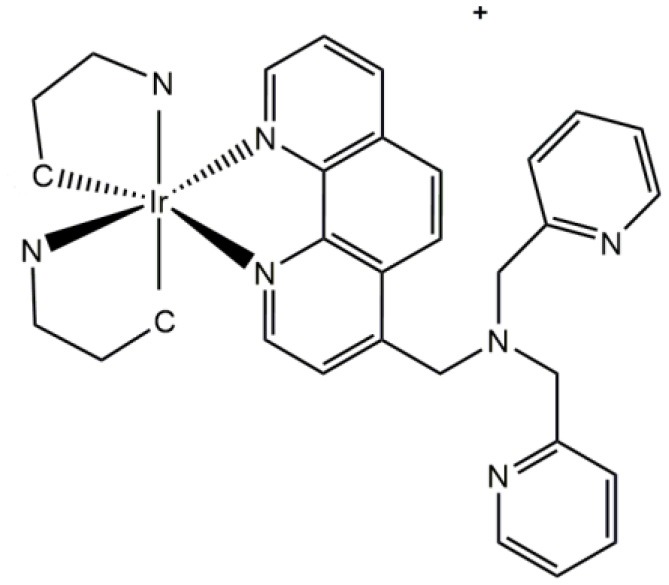	Complexes for phosphorescence sensing of biological metal Ions. This complex is useful to detect Zn (II) ion.	You et al., 2014

Photophysical properties of d^6^ complexes depend directly on the nature of the whole molecule itself, with an emission explained by the triplet metal-to-ligand-charge-transfer (^3^MLCT) as the most important in most molecules (Bonello et al., 2014). ^3^MLCT involves excitation by a photo-induced electron transfer from metal-based orbital (i.e., from Re, Ru, Ir) to a conjugated π-system normally located on an aromatic heterocyclic ligand (often a dinitrogenated ligand) (Long and Wong, 2015). Since excited d^6^ metal-based orbitals must transfer electrons to emit light, d^6^ complexes usually include high-field ancillary ligands (i.e., π acceptors) in order to improve charge transfer (Mitoraj and Michalak, 2010; Lambic et al., 2018; Munoz-Osses et al., 2018). For these reasons, due to low oxidation state of metals [i.e., Re(I), Ru(II), Ir(III)], highly conjugated ligands that can easily accept electronic density are desirable for the development of good fluorescent probes (Cameron et al., 2018; Isik Buyukeksi et al., 2018; Zanoni et al., 2018). In this sense, it is possible to modulate both excitation and emission wavelengths of d^6^ complexes according to the nature of the ligand involved in charge transfer. Thus, the choice of the ligand can directly affect the band gap, impacting, in turn, in the emitting light and remarking the possibility to design complexes with particular luminescent properties (Fernandez-Moreira et al., 2010; Atoini et al., 2017; Ward et al., 2018). All these features contribute to easy excitation and increased quantum yield of d^6^ complexes, in comparison with lanthanide complexes, producing brighter images at lower concentrations and few cytotoxicity, without the need of antennae (Fernandez-Moreira et al., 2010; Thorp-Greenwood, 2012; Thorp-Greenwood et al., 2012).

Besides good luminescent properties, d^6^ complexes must exhibit other additional properties to be suitable for imaging applications in biology. Among these properties, cellular uptake (e.g., by modulating lipophilicity), low cytotoxicity, and specific intracellular localization are crucial, thereby engineering of d^6^ complexes by the presence of different ligands must be explored in order to obtain improved luminescent fluorophores. Since emission comes mainly from the charge transfer between the metal and ligand, exhibiting sensitivity to their electronic levels (Lowry et al., 2004), modifications of the ligands will allow for the designing of new fluorophores with different photophysical properties, but also fluorophores that could be conjugated to other biomolecules (e.g., antibodies) to allow localization-control of the sample.

## Re(I) Tricarbonyl Complexes

As stated, properties exhibited by d^6^ complexes make them attractive for bioimaging applications using fluorescence microscopy (Thorp-Greenwood and Coogan, 2011; Morais et al., 2012). d^6^-based complexes have shown remarkable properties in cellular imaging, especially with epithelial cells, showing specific intracellular localization patterns (Amoroso et al., 2007; Botchway et al., 2008; Li et al., 2011). In particular, Re(I) tricarbonyl complexes have luminescent properties that have long been postulated, but have only been demonstrated relatively recently as being useful as *in vivo* probes (Amoroso et al., 2007, 2008).

**Figure 1:**
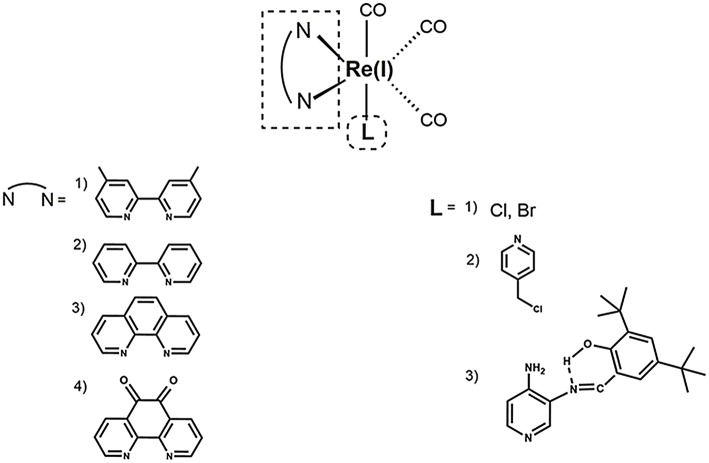
Structural scheme of Re(I) tricarbonyl complexes and their different functional groups [i.e., substituted dinitrogenated ligand (**N**, **N**); ancillary ligand (**L)**]. Some examples of (**N**, **N**) ligands include (1) 4,4′-dimethyl-2,2′-bpy (**dmb**); (2) 2,2′-bipyridine (**bpy**); (3) 1,10-phenanthroline (**phen**); (4) 5,6-dione-1,10-phenanthroline (**dione**). Some examples of ancillary ligands (**L**) include (1) halogens; (2) 3-chloromethylpyridyl; (3) (*E*)-2-{[(3-aminopyridin-4-yl)imino]-methyl}-4,6-di-*tert*-butyl-phenol (a pyridine Schiff base harboring an intramolecular hydrogen bond) (Carreño et al., 2014, 2015a,b, 2017a).

In the first fluorescence studies, Re(I) tricarbonyl complexes with bisquinoline (**bqi**) as substituted trinitrogenated ligand were conjugated to fMLF, a small peptide-based targeting agent used to specifically recognize the formyl peptide receptor (FPR) found in neutrophils (Stephenson et al., 2004), producing a *fac*-[Re(CO)_3_(**bqi**)fMLF]^+^ complex. At low temperatures, fluorescent complexes were located at the same position than the fluorescein-labeled probe, showing that the presence of Re(I) tricarbonyl complexes did not affect neither the recognizing nor localization of fMLF receptor (Stephenson et al., 2004). Although Re(I) **bqi** complexes were the first rhenium species reported as fluorophores for cell imaging, more recent studies demonstrated that dinitrogenated complexes such as 2,2′-bipyridine (**bpy**), 1,10-phenanthroline (**phen**), or derivatives, require longer wavelength excitation compared with **bqi** (i.e., trinitrogenated) ligands, producing low cellular damage but good penetration (Maggioni et al., 2012). In this sense, facial isomers of type *fac*-[Re(CO)_3_(**N**,**N**)**L**]^n^ (where n is 0, +,or -), preferably monocationic complexes where **N**,**N** corresponds to a substituted dinitrogenated ligand and **L** is the ancillary ligand (Figure 1), have been extensively studied due to their photophysical attributes, especially with non-walled eukaryotic cells (Langdon-Jones et al., 2014; North et al., 2015). The relatively lipophilic nature of *fac*-[Re(CO)_3_(**N**,**N**)**L**]^n^ complexes (e.g., they can be dissolved in DMSO) seems also suitable for cell imaging, showing that the choice of the dinitrogenated ligand modulates some photophysical properties (e.g., excitation and emission ranges) (Amoroso et al., 2007), but also some properties as biomarkers (Carreño et al., 2017a).

Most common Re(I) luminescent markers are based on the *fac*-[Re(CO)_3_(**bpy**)**L**]^+^ core, which have been modified in order to develop imaging and sensing agents with diverse properties. These complexes are usually synthesized from parent pentacarbonyl halides, [Re(CO)_5_**X**] (**X** = Cl/Br), to obtain neutral tricarbonyl dinitrogenated halides (e.g., *fac*-[Re(CO)_3_(**bpy**)**X**]) (Kurz et al., 2006; Ranasinghe et al., 2016). In this case, it is important to include a reflux step under an inert atmosphere for 2–3 h (Kurz et al., 2006; Ranasinghe et al., 2016). Nevertheless, more recently a synthesis procedure has been reported that requires only stirring without the need of reflux and inert atmosphere, with a high yield and purity, in only 15–30 min (Carreño et al., 2015b, 2017a). After the synthesis of neutral *fac*-[Re(CO)_3_(**bpy**)**X**, the halide (i.e., **X**) can be substituted by the required ancillary ligand (**L**) to produce the final complexes (i.e., *fac*-[Re(CO)_3_(**bpy**)**L**]^(0, +, or−)^), where total charge depends on the nature of the ancillary ligand **L** (Amoroso et al., 2008; Thorp-Greenwood et al., 2012; Carreño et al., 2016; Carreño et al., 2017a).

Thus, as discussed before, it has been demonstrated that the Re(I) tricarbonyl complexes can be engineered by choosing the ancillary ligand, in order to modulate some properties related to bioimage, including wavelength emission, subcellular localization, and/or cellular uptake. The ancillary ligand can determine lipophilicity, but also other properties such as global charge of the Re(I) tricarbonyl complexes. For example, there are differences in neutral, cationic and anionic forms of Re(I) tricarbonyl complexes. Neutral complexes (e.g., *fac*-[Re(CO)_3_(**N**,**N**)**L**], where **L** is a halogen as substituent) normally exhibit relatively low quantum yield and short lifetime, along with relatively low cellular uptake (Fernandez-Moreira et al., 2010; Carreño et al., 2019). By contrast, cationic *fac*-[Re(CO)_3_(**bpy**)**L**)]^+^ complexes normally present more desirable photophysical properties, including increased lifetime and better quantum yield. In addition, several Re(I) tricarbonyl complexes can be up taken by non-walled eukaryotic cells by passive diffusion, facilitating the staining procedure. Specifically, some cationic lipophilic complexes, e.g., *fac*-[Re(CO)_3_(bpy)(Py-CH_2_OCO(CH_2_)nCH_3_)]^+^ (Py = pyridine; *n* = 6, 12, 16), co-localize in internal membranes of organelles, or other lipophilic cytoplasmic structures (Stufkens and Vlcek, 1998; Coogan and Fernandez-Moreira, 2014). On the other hand, anionic complexes (e.g., *fac-*[Re(CO)_3_((SO_3_-phenyl)_2_-**phen**)(Py-R)]^−^(Py: pyridine; R: H, CH_2_OH, CH_2_OCOC_13_H_27_) accumulate only on the outer face of the plasma membrane, or show no cellular uptake at all despite the presence of very lipophilic substituents (Amoroso et al., 2007), showing that a suitable combination of both the dinitrogenated and the ancillary ligand must be performed in order to develop an adequate biomarker.

Besides contributing to the global charge of the complex, ancillary ligands can also modulate other properties, showing this moiety was not as irrelevant, as previously suggested (Sacksteder et al., 1990). For instance, a group of *fac*-[Re(CO)_3_(**N**,**N**)**L**]^+^ (where **N**,**N** is **bpy** or **phen** derivatives) complexes, where **L** corresponds to a highly lipophilic series including esters of 3-hydroxymethylpyridine, i.e., Py-3-CH_2_O_2_CR′ (Py = pyridine; R′ = octyl, merystyl, or steryl), were tested as biomarkers in both liposomes and *Spironucleus vortens*, a unicellular eukaryotic fish parasite related to *Giardia* spp. (Amoroso et al., 2007). Results showed that lipophilic *fac*-[Re(CO)_3_(**subs-bpy**)**L**]^+^ complexes (where **subs-bpy** is a substituted **bpy**, and **L** is a highly hydrophobic ligand) were associated to cell membranes, internal membranes of organelles and cell debris. Interestingly, the neutral compound *fac*-[Re(CO)_3_(**phen**)**Cl**] was mainly found in aqueous fractions and not in membranes, suggesting that high lipophilicity and/or a cationic nature are required for an efficient uptake and staining, as stated above (Amoroso et al., 2007). Thus, apparently, the use of lipophilic complexes could lead to an improved luminescent staining in cells. Nevertheless, highly lipophilic *fac*-[Re(CO)_3_(**N**,**N**)**L**]^+^ exhibit cytotoxicity, mainly due to membrane disruption that ultimately led to cell lysis. In this sense, it has been stated that *fac*-[Re(CO)_3_(**N**,**N**)**L**]^+^ complexes are not toxic *per se*, but toxicity can be a problem depending on the chosen ancillary ligand (**L**) (Amoroso et al., 2007; Hallett et al., 2018), indicating that experimental research is necessary to establish biocompatibility in each case. Besides cytotoxicity, ancillary ligands can also modify other properties in Re(I) tricarbonyl complexes involved in bioimaging. In this regard, it was stated that chloride used as an ancillary ligand promotes high cell-depending photobleaching when used in these complexes (Amoroso et al., 2007). Nevertheless, other similar neutral Re(I) tricarbonyl complexes harboring bromide as ancillary ligand, instead of chloride, seem to be resistant to photobleaching, exhibiting an efficient stain of different cell models and reinforcing the need of an experimental approach in each case to assess the complexes as biomarkers (Carreño et al., 2017a).

Ligands can also modify other properties with potential use in biological applications. For instance, alkoxy bridged binuclear Re(I) tricarbonyl complexes containing long alkyl chains with photoisomerizable 4-(1-naphthylvinyl) pyridine ligand (1,4-NVP) enhance its fluorescent emission in the presence of β-amyloid fibrils, exhibiting a potential in Alzheimer's disease diagnosis (Sathish et al., 2014), remarking an eventual use of Re(I) tricarbonyl complexes as novel differential probes and opening new windows in medical approaches.

## Re(I)-Based Fluorophores for Walled Cells

As stated above, several fluorophores have been extensively studied regarding their use for non-walled cells (e.g., cell lines, usually epithelial cells). However, in the last years, the use of Re(I)-based fluorophores in walled cells, in particular bacteria and fungi, have been explored. Many pathogens correspond to bacteria and fungi (yeasts and molds). In addition, these organisms have been increasingly used in innumerable biotechnological applications, underlining the importance of developing new tools for their study, such as the design of new fluorophores. Nevertheless, development of Re(I)-based fluorophores for walled cells has encountered some troubles. Both bacteria and fungi possess a rigid structure found in their respective envelop, called cell wall (Sanz et al., 2017; Caveney et al., 2018). The presence of the cell wall could impair incorporation of foreign molecules, including Re(I)-based complexes, as previously proposed (Amoroso et al., 2007, 2008). According to our experience, the development of Re(I)-based fluorophores for walled cells is possible, but requires systematic experimentation to find suitable ligands surrounding the metallic core. Although much of the evidence showing adequate Re(I)-based fluorophores for walled cells is empirical, we can list some common features that favor their use for these kind of cells.

With respect to substitutions in the denitrogenated ligand in *fac*-Re(I)(CO)_3_(**N,N**)**X** complexes (where **N,N** is a denitrogenated ligand and **X** is a halide), apparently larger substituents seem to impair the properties as fluorophores in yeasts, showing, for instance, that *fac*-Re(I)(CO)_3_(**2,2′-bpy**)**Br** exhibit better staining than *fac*-Re(I)(CO)_3_(**4,4′-diethanoate-2,2′-bpy**)**Br** (Carreño et al., 2017a). Interestingly, it has been stated that some dinitrogenated ligands alone (e.g., 1,10-phenanthroline or derivatives) are highly cytotoxic toward different cell types, including walled cells such yeasts and bacteria (Coyle et al., 2003; Roy et al., 2008; Kaplanis et al., 2014; Carreño et al., 2019). Nevertheless, it has been shown that, when these dinitrogenated ligands are coordinated through their two nitrogens with the metal, cytotoxicity is strongly diminished (Carreño et al., 2017a; Carreño et al., 2019). Low cytotoxicity is fundamental for fluorophores, indicating that the most common dinitrogenated ligands, such as 1,10-phenanthroline derivatives, can be used to develop luminescent fluorophores for walled cells.

Regarding the total charge, a cationic nature of *fac*-[Re(CO)_3_(**2,2′-bpy**)**L**)]^+^ complexes is desirable for the generation of luminescent fluorophores, even for walled cells, due to advantageous photophysical properties and improved uptake (Coogan and Fernandez-Moreira, 2014; Carreño et al., 2016, 2017a; Carreño et al., 2019; Carreño et al., 2019a). This is apparently true for other d^6^-based complexes used to stain walled cells. For instance, prototypical *cis*-Ru(II)(**N,N**)$3_{2}^{+}$ complexes (where **N,N** is a dinitrogenated ligand) were reported to be useful to stain yeasts (Carreño et al., 2019b). However, it is necessary to be cautious since highly charged cationic d^6^-based complexes are apparently unable to penetrate walled cells. This is the case for ruthenium red ([(NH_3_)_5_Ru(II)–O–Ru(II)(NH_3_)_4_–O–Ru(II)(NH_3_)_5_]^6+^), used as a dense material to stain extracellular components in yeasts, a compound that is unable to penetrate cells (Farrington and Sannes, 2015).

As stated above, the choice of both the dinitrogenated ligand and the total charge of the complex are important to generate a suitable fluorophores for walled cells. In this context, the ancillary ligand also plays a relevant role, albeit the choice of the right ligand is not trivial. Amoroso et al. tested different ancillary ligands in *fac*-Re(I)(CO)_3_(**2,2′-bpy**)**L**^+^, where **L** is a *meta*-substituted pyridine with ester aliphatic chains (from 6 to 16 carbons), and found that these complexes were toxic for different cell kinds, inducing cell disruption and affecting the image obtained by fluorescence microscopy (Amoroso et al., 2007). Later, Amoroso et al. explored a different ancillary ligand using the same *fac*-Re(I)(CO)_3_(**2,2′-bpy**)**L**^+^ core, but using 3-chloromethylpyridyl instead of *meta*-substituted pyridine with ester aliphatic chains (from 6 to 16 carbons) as **L**. Although these new complexes were significantly less toxic than complexes harboring long aliphatic chains, producing better images, the use of 3-chloromethylpyridyl as ancillary ligand seems to be useful only for non-walled cells (i.e., breast cancer cell line). By contrast, when this same complex was used to stain yeasts, poor results were obtained, showing only a small proportion of cells that retained the fluorophore (Amoroso et al., 2008). These findings remark the fact that, although the cationic nature has been proposed as being important for the uptake by non-walled cells (Langdon-Jones et al., 2014), it is also necessary to find suitable ancillary ligands to allow uptake by walled cells. In this way, it has been reported that one particular kind of pyridine Schiff base harboring an intramolecular hydrogen bond is useful to act as ancillary ligands to generate Re(I) complexes useful to stain walled cells (Carreño et al., 2015b, 2016, 2017a, 2019a). Schiff bases are aldehyde- or ketone-like compounds, where the carbonyl group is replaced by an azomethine (–C=N–) group (Da Silva et al., 2011). In general, Schiff bases have been used for diverse applications, including antimicrobial compounds, due to their high cytotoxicity against bacteria or fungi (Jarrahpour et al., 2007; Justin Dhanaraj and Sivasankaran Nair, 2009). At a first sight, an ancillary ligand exhibiting cytotoxic activity is not desirable. Nevertheless, it has been established that pyridine Schiff bases harboring an intramolecular hydrogen bond depend on the non-coordinated nitrogen found in the pyridine ring to exert their antimicrobial activity (Carreño et al., 2015a,b, 2018a,b). Considering that coordination of Re(I) core occurs through the pyridine nitrogen in this kind of Schiff bases, the resulting *fac*-Re(I)(CO)_3_(**N,N**)(**pyridine Schiff Base**)^+^ complexes exhibit lower cytotoxicity for walled cells, when compared with the respective free ancillary ligand (Carreño et al., 2015a,b, 2016, 2017a,b, 2018a,b). More importantly, *fac*-Re(I)(CO)_3_(**N,N**)(**pyridine Schiff Base**)^+^ complexes are useful to observe walled cells, including bacteria and fungi, through fluorescence microscopy. Thus, an efficient staining can be achieved with a simple protocol, with short incubation times (15–30 min), at 37°C, and in absence of an additional permeabilizer agent (Carreño et al., 2016, 2017a). Interestingly, these same complexes were also useful to stain non-walled cells (i.e., epithelial cell line), but only after long incubations periods (48–72 h) (Carreño et al., 2016), suggesting that these Re(I) complexes could be considered as being especially designed for walled cells.

**Figure 2:**
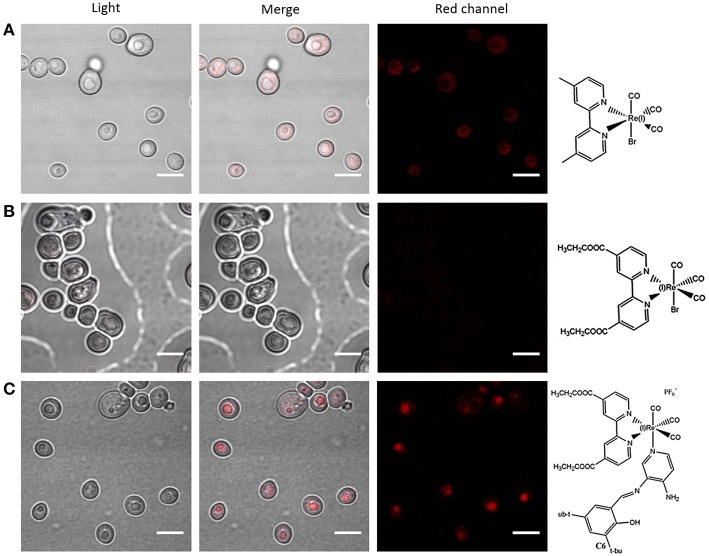
Effect of ligands in the use of *fac*-Re(I)(CO)_3_(**N,N**)**L**^(0, +)^ complexes in walled cells (yeasts). Fluorescence confocal microscopy images of *Candida albicans* (yeasts) stained with *fac*-Re(I)(CO)_3_(**4,4′-dimethyl-2,2′-bpy**)**Br (A)**, *fac*-Re(I)(CO)_3_(**4,4′-diethanoate-2,2′-bpy**)**Br (B)**, or *fac*-Re(I)(CO)_3_ (**4,4′-diethanoate-2,2′-bpy**) [**(*E*)-2-((3-amino-pyridin-4-ylimino)-methyl)-4,6-di-*tert*-butylphenol**]^+^ **(C)** were compared. “Red channel” corresponds to excitation of 405 nm and emission collected in a range of 555 to 625 nm. In all cases, microorganisms were observed fresh, immobilized with 1% agarose, using a 100× objective. DMSO alone was used to set the detection threshold (not shown). White bars represent 5 μm. The complete protocol for staining and other examples of how ligand choice impacts on staining properties of d^6^ complexes were previously reported (Carreño et al., 2016, 2017a, 2019b).

A combination of different features in the Re(I)-based fluorophores, such as a cationic nature, a dinitrogenated ligand and a suitable ancillary ligand (e.g., as a pyridine Schiff base such as (*E*)-2-((3-amino-pyridin-4-ylimino)-methyl)-4,6-di-*tert*-butylphenol)) (Carreño et al., 2016, 2017a) are useful to develop new fluorophores for walled cells. Remarkably, small modifications in the nature of these components, such as the substituent groups in the dinitrogenated ligand, can even allow for obtaining differential fluorophores. For instance, *fac*-Re(I)(CO)_3_(**2,2′-bpy**)(**(*E*)-2-((3-amino-pyridin-4-ylimino)-methyl)-4,6-di-*tert*-butylphenol)**)^+^ can differentially stain bud-like structures when used to stain *Candida albicans* or *Cryptococcus* spp. (yeasts). By contrast, a small change in the dinitrogenated ligand, with the addition of a polar group (*fac*-Re(I)(CO)_3_(**4,4′-diethanoate-2,2′-bpy**)(**(*E*)-2-((3-amino-pyridin-4-ylimino)-methyl)-4,6-di-*tert*-butylphenol)**^**+**^) produces a fluorophore that specifically stains the cell nucleus in these same fungi (Carreño et al., 2016, 2017a). This phenomenon was also observed with other d^6^-based fluorophores, where *cis*-Ru(II)(**4,4′-diethanoate-2,2′-bipyridine**)$3_{2}^{+}$ was useful to stain yeasts, remaining retained in a structure consistent with the nucleus, whereas *cis*-Ru(II)(**1,10-phenanthroline**)$3_{2}^{+}$ was retained in a peripheric structure of the yeast, probably cell membrane or cell wall (Carreño et al., 2019b). This evidence supports that relatively small substitutions, not necessarily involving long aliphatic chains, are enough to change the properties of other d^6^-based fluorophores, including Re(I) and Ru(II) complexes. In this regard, potential intermolecular interactions that these substituents could form with biological systems, is a fundamental property to be considered in the development of d^6^-based differential luminescent dyes (Carreño et al., 2017a). Some examples of how ligands affect staining properties of Re(I) complexes in walled cells are shown in Figure 2. High versatility of the d^6^-based fluorophores will plausibly allow the generation of diverse biological probes, even in the absence of other moieties aimed to provide specificity, such as antibodies.

Regarding filamentous fungi (mold, walled cells), Re(I) complexes harboring a pyridine Schiff base have been shown to be useful to stain both spores and hyphae of *Botrytis cinerea*, a ubiquitous necrotrophic filamentous fungal pathogen causing the “gray mold” disease in a wide range of plants. Since hyphae and conidia from *Botrytis cinerea* present a dynamic multilayer cell wall that varies the composition during normal growth (Cantu et al., 2009), it is difficult to develop suitable fluorophores to stain these structures. Recently, it has been reported that cationic Re(I) complexes with a dinitrogenated ligand and a pyridine Schiff base as ancillary ligand (e.g., *fac*-Re(I)(CO)_3_(**2,2′-bpy**)(**(*E*)-2-((3-amino-pyridin-4-ylimino)-methyl)-4,6-di-*tert*-butylphenol)**^+^) were useful to stain *Botrytis cinerea* structures, including conidia and juvenile hyphae. In that work, a new protocol was proposed as incubation at higher temperatures (65°C) can be useful to stain this kind of fungal structures. Furthermore, evidence of selective staining of living conidia was provided, opening a new focus for the generation of Re(I)-based fluorophores with potential use for vital staining (Carreño et al., 2019a).

## Conclusion

Use of Re(I)-based complexes as fluorophores is increasingly gaining attention. Development of new applications in walled cells, such as bacteria and fungi, has underlined that systematic research of the best molecular features is fundamental to engineer new, improved fluorophores. Accordingly, *fac*-Re(I)(CO)_3_(**N,N**)**L**^+^ complexes should fulfill some desirable structural features:

1. Charge: Monocationic nature.
2. **N,N**: the presence of a **bpy** or **phen** with relatively small substituents (e.g., methyl, ethyl ester). Changes in these substituents can produce complexes for differential staining.
3. Ancillary ligand: An ancillary ligand lacking long aliphatic chains but preferentially presenting groups favoring the formation of hydrogen bonds. Hydrogen bonds potentially could improve interactions with biomolecules found in biological systems, improving retention.

A combination of these three features provides high plasticity to develop new Re(I) complexes with specific properties, adapted for a particular purpose regarding the generation of fluorophores for walled cells. It also important to remark that it is necessary to establish a suitable staining protocol since, depending on the organisms, increasing temperature or incubation time could greatly improve results.

Finally, it is possible to conclude that d^6^-based complexes exhibit a high versatility, allowing the development of new molecules for diverse applications, including fluorophores especially designed for walled cells, showing low cytotoxicity, cellular uptake and differential staining properties.

## Author Contributions

CO contributed with bibliographic research of eukaryotic, non-walled cells, and critical reading. AC contributed with the whole chemical section (Table 1), general discussion, review organization, and paper writing. RP contributed with bibliographic research of molds and critical reading. FL contributed with critical reading and Figure 1. RA-P contributed with critical reading, with emphasis on rhenium chemistry. MG contributed with critical reading, with emphasis on chemical properties. JF contributed with the whole biological section (Table 1; Figure 2), general discussion, review organization, and paper writing.

### Conflict of Interest Statement

The authors declare that the research was conducted in the absence of any commercial or financial relationships that could be construed as a potential conflict of interest.
